# Predictive value of percutaneous peripheral arterial compliance T in left ventricular diastolic function with coronary artery disease

**DOI:** 10.3389/fcvm.2024.1366072

**Published:** 2024-11-08

**Authors:** Wenhao Zhang, Guoliang Liang, Liang Lv, Xinxin Gu, Qiong Zhang, Ankang Liu, Jiangwei Ma

**Affiliations:** ^1^ Department of Cardiology, Jinzhou Medical University, Jinzhou, Liaoning, China; ^2^ Department of Cardiology, Shanghai Fengxian District Central Hospital, Shanghai, China; ^3^ Department of Cardiology, Anhui University of Science and Technology, Huainan, Anhui, China; ^4^ Department of Cardiology, Xi'an Central Hospital, Xi’an, Shaanxi, China

**Keywords:** arterial elasticity index, left ventricular dysfunction, coronary artery disease, heart failure, ejection fraction

## Abstract

**Background:**

Diastolic dysfunction emerges early in patients with cardiac insufficiency and is prevalent, underscoring the importance of its early identification and intervention in the prevention of heart failure. The study leverages the convenience and accuracy of measuring peripheral arterial sclerosis to facilitate the early detection of diastolic dysfunction, which is instrumental in mitigating and delaying the onset and progression of heart failure, holding significant clinical relevance.

**Methods:**

This research enrolled 556 patients suspected of experiencing Acute Coronary Syndrome (ACS) and stratified them into Groups A, B, C, and D according to the severity of coronary artery stenosis. The diastolic function of the left ventricle was indicated by the relaxation time constant, denoted as T value, which measures the temporal span from the peak rate of left ventricular pressure rise (dp/dt) to the end-diastolic pressure.

**Results:**

The T value of the left ventricle demonstrated a significant correlation with the Gensini Score and the T values across various peripheral arteries (*P* < 0.01). Pearson correlation analysis showed that the average value of peripheral arterial compliance indices in Group C and the average value of peripheral arterial compliance indices in Group D had a significant correlation with LV-T. At the same time, linear analysis of the average values of peripheral arterial compliance indices in both groups revealed that the average compliance indices in Groups C and D had a linear correlation with their LV-T (*P* < 0.05).When coronary artery stenosis exceeds 50%, the changes in peripheral arterial T values are significantly correlated with changes in LV-T.

**Conclusions:**

When coronary artery stenosis exceeds 50%, there is a decrease in peripheral artery compliance, showing a positive correlation with changes in left ventricular diastolic function. Measuring this compliance might offer an early diagnostic tool for assessing diastolic function.

## Introduction

The menace of cardiovascular disease to human health has intensified with the aging of the population and the ongoing rejuvenation of the ailment (1). A 2020-based census in the United States projects a 31.1% increase in the prevalence of cardiovascular disease by 2060 compared to 2025 (2). Concurrently, research indicates that cardiovascular disease will pose a significant challenge to human health (3, 4). Among these challenges, heart failure assumes a central position. Presently, approximately 64.3 million people worldwide grapple with heart failure (5), and this number continues to rise due to factors such as an aging population, sluggish global population growth, and improved survival rates post-diagnosis (6).

Heart failure is not a single pathological diagnosis, but a clinical syndrome consisting of cardinal symptoms (e.g., breathlessness, ankle swelling, and fatigue) that may be accompanied by signs (e.g., elevated jugular venous pressure, pulmonary crackles, and peripheral oedema). It is due to a structural and/or functional abnormality of the heart that results in elevated intracardiac pressures and/or inadequate cardiac output at rest and/or during exercise. In accordance with the “2021 ESC Guidelines for the Diagnosis and Treatment of Acute and Chronic Heart Failure,” heart failure can be categorized based on the left ventricular ejection fraction into three classifications: Heart Failure with Reduced Ejection Fraction (HFrEF), Heart Failure with Mildly Reduced Ejection Fraction (HFmrEF), and Heart Failure with Preserved Ejection Fraction (HFpEF) (7). The initial manifestation of heart failure involves a reduction in diastolic function while maintaining normal systolic function. Consequently, some patients with Heart Failure with Preserved Ejection Fraction are prone to being overlooked. It is estimated that approximately 76% of unrecognized heart failure cases involve patients with preserved left ventricular ejection fraction (8). Hence, there is significant importance placed on the prevention and diagnosis of HFpEF. Abnormal arterial stiffness emerges in the early stages of heart failure, and diminished arterial compliance elevates left ventricular afterload while reducing coronary perfusion, contributing to cardiac dysfunction (9). Consequently, the clinical significance lies in the ability to predict or diagnose HFpEF through alterations in peripheral arterial compliance.

In our investigation, we meticulously assessed peripheral arterial compliance and left ventricular diastolic function using precise invasive testing. The aim was to accurately evaluate peripheral arterial compliance and its correlation with cardiac diastolic function. By doing so, we sought to identify and advance the therapeutic window at the earliest possible stage, thereby holding significant clinical relevance for the early diagnosis of ejection fraction-preserved heart failure or slowing down its onset and progression.

## Materials and methods

### Research population

**Figure 1:**
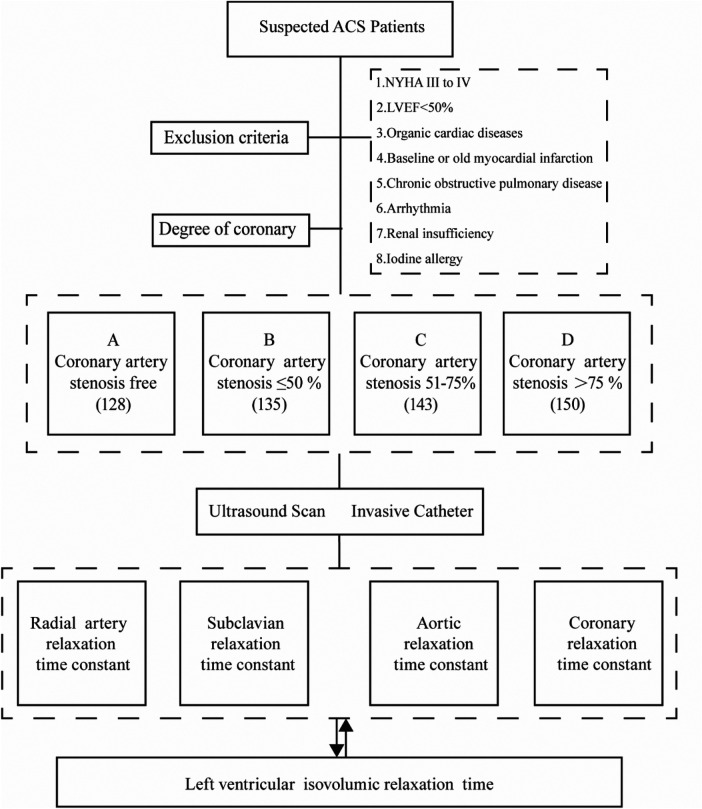
Study design and patients. **(A)** Coronary artery stenosis free, **(B)** Coronary artery stenosis less than 50%, **(C)** Coronary artery stenosis of 51%–75%, **(D)** Coronary artery stenosis greater than 75%.

This study conducted a retrospective analysis on 556 patients suspected of Acute Coronary Syndrome (ACS) at the South Hospital of the Sixth Affiliated Hospital of Shanghai Jiaotong University, spanning from June 2020 to May 2023 (Figure 1). All patients underwent coronary arteriography during this period. The cohort comprised 264 males and 292 females. Based on the degree of coronary artery stenosis observed, the patients were categorized into four groups: Group A (no coronary artery stenosis), Group B (coronary artery stenosis less than 50%), Group C (coronary artery stenosis between 51% and 75%), and Group D (coronary artery stenosis greater than 75%).

Inclusion criteria: (1) Meeting the diagnostic criteria for ACS; (2) Patients with complete clinical information.

Exclusion criteria: (1) patients with heart failure in the New York Heart Association (NYHA) cardiac function class III to IV or above; (2) cardiac ultrasound suggestive of cardiomyopathy, heart valve disease, myocardial amyloidosis, congenital heart disease, pericardial disease, ventricular wall tumor, and other organic cardiac diseases; (3) patients with LVEF < 50%; (4) patients with baseline myocardial infarction or old myocardial infarction; (5) patients with chronic obstructive pulmonary disease; (6) patients with arrhythmia; (7) patients with renal insufficiency. 50%; (8) history of iodine allergy.

All enrolled patients signed an informed consent form and were approved by the Ethics Committee (full name: Medical Ethics Committee of Shanghai Fengxian District Central Hospital) (reference number: 2014-KY-06).

### Clinical information collection

We collected essential information from all patients, encompassing gender, age, history of hypertension, diabetes mellitus, and smoking. Additionally, we gathered data on blood biochemical indicators of the patients: blood lipids (including triglycerides, total cholesterol, High-Density Lipoprotein (HDL), Low-Density Lipoprotein (LDL), blood creatinine, Troponin I, and B-type natriuretic peptide (BNP).

### Echocardiogram

We utilized a PHILIPS 7C ultrasonic diagnostic ultrasound machine (No. 32904334) equipped with a probe frequency of 2.5–3.5 MHz. Patients were positioned in the left lateral stance, and imaging included parasternal left ventricular long-axis views, left ventricular short-axis views, as well as apical four-chamber and two-chamber cardiac views. Indicators reflecting left ventricular (LV) compliance and filling status were quantified, encompassing left ventricular end-diastolic volume (LVEDV), left ventricular end-systolic internal diameter (LVESD), end-diastolic internal diameter (LVEDD), and left ventricular ejection fraction (LVEF).

The aforementioned procedures were conducted by experienced sonographers with the rank of associate chief physician or higher, performing M-mode ultrasound measurements, including the deceleration time (DT) of the peak early diastolic flow of the mitral valve and the peak ratio of mitral annular motion velocity (E/e’). Each of the above measurements was taken three times, and the values were averaged to enhance accuracy (10).

### Peripheral arterial ultrasonography

Carotid-femoral pulse wave conduction velocity (cfPwv) was assessed using a PHILIPS 7C ultrasound diagnostic machine (No. 32904334) equipped with a 12–3 MHz probe and a randomly configured digital management system (e-DMS). Patients were positioned without pillows, lying on their backs. The cfPwv pressure receptor was strategically placed at the right carotid and femoral arteries’ most prominent pulsation point, and the surface distance between the two locations was precisely measured and recorded in the computer (11).

These procedures were autonomously conducted by a sonographer of deputy director or higher rank. The image measurements were carried out in the presence of two professional deputy director physicians, and if necessary, measurements could be repeated up to three times, with the average value taken for accuracy.

Preparations for the examination operation involved certain measures. Within 24 h before the examination, the examinee refrained from using vasoactive drugs, avoided strenuous activities, maintained emotional stability, and abstained from strong tea, coffee, tobacco, and alcohol for the preceding 6 h. The indoor temperature was kept between 22 and 26℃, and the examinee assumed a flat position without a pillow.

### Coronary angiography, left ventriculography, and peripheral arterial pressure measurement

Coronary Angiography (CAG): For CAG, a sophisticated C-arm digital subtraction x-ray system, paired with a multidirectional physiological recorder (Model: AXIOM Artis Zee Celling, Device serial number: 147191, Device identification number: 720-939180), was employed. Typically, the patient's right radial artery served as the puncture site, and following routine disinfection, a sterile towel was used, sterile gloves were donned, and local infiltration anesthesia with 1% lidocaine was administered. After successful puncture of the radial artery using Seldinger's method, a 6F radial artery sheath was inserted along the ultraslip guidewire, ensuring the removal of air bubbles and injecting a solution consisting of 5 ml of 1% lidocaine, 0.3 mg of nitroglycerin, and 3,000 IU of heparin. Choosing the optimal contrast position, a 5F multi-purpose contrast catheter was employed for contrast, guided by two experienced cardiac catheterization deputy directors, adhering to the cardiology standards in the United States (12, 13). The modified Gensini score was utilized to assess the degree of coronary stenosis upon completion of coronary angiography and hemodynamics. Following these procedures, left ventriculography was performed.

Left ventriculography: In this study, patients were positioned at a 30° right anterior oblique angle and imaged at 50 frames/s using a 6F pigtail catheter and a high-pressure syringe for rapid injection of iodixanol contrast agent. x-ray machine software was used for replay and measurement. The electrocardiogram and left ventricular pressure curve were continuously and synchronously recorded for at least 5 consecutive cardiac cycles.

Peripheral arterial pressure measurement:(Maximum velocity of radial artery rise, R + dp/dtmax)、 Maximum velocity of radial artery descent(R-dp/dtmax)、Maximum speed of aortic rise(A + dp/dtmax)、Maximum velocity of aortic descent(A-dp/dtmax)、Maximum velocity of ascending right subclavian artery (S + dp/dtmax)、Maximum velocity of descent of the right subclavian artery(S-dp/dtmax)、Maximum rate of coronary pressure rise(C + dp/dtmax)、Maximum rate of coronary pressure drop(C-dp/dtmax)、Radial artery relaxation time constant(R-T)、Right Subclavian relaxation time constant (S-T)、Aortic relaxation time constant(A*-T)、Coronary relaxation time constant(C*-T).

Measurement of indices of left ventricular diastolic function: +dp/dtmax of systolic pressure (LV + dp/dtmax), -dp/dtmax of diastolic pressure (LV-dp/dtmax), and Left ventricular time constant (LV-T) based on first-order differential curves of the left ventricular pressure curve (14).

$$\;\;\text{①}\;\;\;\;T=-\frac{1}{A}\;\;\;\text{②}\;\;\;\;A=\frac{Ln\Delta P}{t}\;\;\mathrm{Normal}\;\mathrm{Value}<40\mathrm{ms}$$

### Statistical methodology

Statistical analyses were processed with SPSS 26.0 statistical software. Measurement data were expressed as mean ± standard deviation, and comparisons between groups were made by One- ANOVA; count data were expressed as percentage, and comparisons between groups were made by chi-square test. Pearson correlation analysis and Linear regression model were used to analyze the relationship between multiple risk factors and peripheral arterial pressure curves with LV diastolic function.

## Results

In this investigation, a total of 556 patients with suspected ACS were enrolled and classified based on the degree of coronary stenosis into four groups: Group A (no significant coronary stenosis) with 128 cases, Group B (coronary stenosis not exceeding 50%) with 135 cases, Group C (coronary stenosis ranging from 51%–75%) with 143 cases, and Group D (coronary stenosis >75%) with 150 cases. When comparing baseline data among the four groups, it was noted that the average age of patients in Groups A, B, C, and D was 59 years, with a male proportion of 47%. The differences in age, gender, smoking, and the prevalence of hypertension among the four groups were not statistically significant (*P* > 0.05). There was no statistically significant difference observed in HDL, LDL, Cholesterol, Triglycerides, Creatinine, Troponin I, BNP, and other indices among the four groups (*P* > 0.05). However, the Gensini score, reflecting the degree of coronary artery stenosis, exhibited a noteworthy variation among the groups, with a statistically significant difference (*P* < 0.001). Group D notably presented a significantly higher Gensini score compared to the remaining three groups, and the scores of Groups B, C, and D increased proportionally with the escalation in the degree of coronary artery stenosis, providing further validation for the heightened severity of coronary artery stenosis in Group D patients (Table 1).

**Table 1:** Baseline characteristics of patients with suspected ACS.

	A (*n* = 128)	B (*n* = 135)	C (*n* = 143)	D (*n* = 150)	*P*-value	95% CI
Male(%)	60 (47)	69 (51)	67 (47)	68 (45)	0.793	–
Age(y)	56.77 ± 12.61	59.11 ± 11.04	59.13 ± 11.27	59.91 ± 10.85	0.129	(−5.11,0.42)
Smoker(%)	35 (27)	34 (25)	39 (27)	36 (24)	0.897	–
Hypertension(%)	70 (55)	77 (57)	78 (55)	88 (59)	0.877	–
Diabetes(%)	34 (27)	48 (36)	48 (34)	50 (33)	0.431	–
HDL(mmol/L)	1.48 ± 0.37	1.41 ± 0.35	1.41 ± 0.36	1.40 ± 0.34	0.239	(−0.21,0.15)
LDL(mmol/L)	2.73 ± 0.52	2.73 ± 0.63	2.73 ± 0.73	2.87 ± 0.66	0.148	(−1.48,0.16)
Cholesterol(mmol/L)	4.12 ± 0.84	4.34 ± 1.13	4.14 ± 1.14	4.25 ± 1.18	0.286	(−0.47,0.04)
Triglycerides(mmol/L)	1.44 ± 0.49	1.37 ± 0.46	1.39 ± 0.43	1.44 ± 0.44	0.541	(−0.04,0.18)
AST(U/L)	24.63 ± 7.23	22.79 ± 6.74	24.88 ± 7.56	24.45 ± 6.97	0.066	(−0.11,.3.57)
ALT(U/L)	24.05 ± 7.34	23.64 ± 8.40	23.72 ± 8.84	22.87 ± 7.42	0.645	(−1.53,2.36)
Creatinine(*μ*mol/L)	62.45 ± 15.00	65.94 ± 11.98	65.09 ± 11.89	65.17 ± 12.64	0.142	(−6.31,−0.36)
cTnI(ng/ml)	0.006 ± 0.004	0.006 ± 0.003	0.005 ± 0.003	0.006 ± 0.003	0.433	(−0.004,0.001)
BNP(Pg/ml)	43.08 ± 20.00	42.71 ± 23.23	41.53 ± 23.47	39.31 ± 21.23	0.470	(−4.98,5.71)
Gensini score	0^a^	4.61 ± 1.94^b^	12.37 ± 2.62^c^	50.50 ± 13.00^d^	<0.001	(48.86,52.14)

A: Coronary artery stenosis free, B: Coronary artery stenosis less than 50%, C: Coronary artery stenosis of 51–75%, D: Coronary artery stenosis greater than 75%.Values are mean ± standard deviation or n(%).

HDL, high-density lipoprotein; LDL, low-density lipoprotein; AST, Aspartate aminotransferase; ALT, Alanine aminotransferase; cTnI, cardiac troponin I; BNP, brain natriuretic peptide.

^a,b,c,d^ are markers of difference between the groups, the same letter indicates that the difference between the two groups is not statistically significant, and different letters indicate that the difference is statistically significant.95%CI: Confidence Interval.

### Deterioration of left ventricular diastolic function with increased coronary artery stenosis

Echocardiographic data were gathered from the four patient groups, and a one-way ANOVA analysis unveiled that indices such as cfPwv, DT, LVEDV, LVESD, LVEDD, and E/e’ demonstrated an incremental pattern corresponding to the severity of coronary artery stenosis. Significantly, cfPwv, LVEDV, LVEDD, and E/e’ indices exhibited notable differences among the groups, with statistical significance (*P* < 0.001). Simultaneously, upon comparing the LVEF among patients in each group, it was discerned that the LVEF of patients in Groups B, C, and D was notably lower than that of Group A. A one-way ANOVA analysis further highlighted a significant difference among the four groups, and this distinction was statistically significant (*P* < 0.001). Essentially, this signifies a reduction in LVEF with an escalation in the degree of coronary stenosis. Furthermore, through one-way ANOVA analysis of patients’ left ventricular diastolic function indices, DT, and LVESD, it was determined that there was no statistically significant difference between Groups A and B (*P* > 0.05). However, a significant difference was observed between Groups C and D, and this dissimilarity proved to be statistically significant (*P* < 0.001) (Table 2).

**Table 2:** Echocardiographic detection of suspected ACS patients.

	A (*n* = 128)	B (*n* = 135)	C (*n* = 143)	D (*n* = 150)	*P*-value	CI
cfPwv	10.53 ± 0.64^a^	10.80 ± 0.71^b^	13.27 ± 0.52^c^	13.99 ± 0.59^d^	<0.001	(3.31,3.60)
LVEF	69.00 ± 3.11^a^	68.16 ± 3.63^b^	66.56 ± 3.76^c^	60.55 ± 4.19^d^	<0.001	(−9.33,−7.57)
DT	199.16 ± 25.02^a^	205.64 ± 27.07^a^	230.86 ± 26.17^c^	247.39 ± 27.82^d^	<0.001	(41.95,54.52)
LVEDV	143.95 ± 2.88^a^	145.85 ± 3.15^b^	147.64 ± 2.81^c^	151.22 ± 3.06^d^	<0.001	(6.57,7.98)
LVESD	39.78 ± 2.17^a^	40.10 ± 2.44^a^	41.23 ± 2.48^c^	41.91 ± 2.81^d^	<0.001	(1.54,2.72)
LVEDD	45.32 ± 3.03^a^	47.23 ± 4.53^b^	48.57 ± 3.91^c^	50.57 ± 3.67^d^	<0.001	(4.34,6.15)
E/e’	9.28 ± 0.80^a^	10.14 ± 0.82^b^	13.40 ± 1.00^c^	14.62 ± 0.99^d^	<0.001	(5.11,5.54)

A: Coronary artery stenosis free, B: Coronary artery stenosis less than 50%, C: Coronary artery stenosis of 51–75%, D: Coronary artery stenosis greater than 75%.Values are mean ± standard deviation; cfPwv, carotid-femoral pulse wave velocity; LVEF, left ventricular ejection fractions; DT, peak E deceleration time; LVEDV, Left ventricular end-diastolic volume; LVESD, left ventricular end systolic dimension; LVEDD, left ventricular end-diastolic diameteR; E/e’, ratio of peak early mitral valve diastolic flow to mitral annular motion velocity.

^a,b,c,d^ are markers of difference between the groups, the same letter indicates that the difference between the two groups is not statistically significant, and different letters indicate that the difference is statistically significant. 95%CI: Confidence Interval.

Furthermore, during transradial artery puncture coronary angiography, data on peripheral arteries, left coronary artery, and left ventricular pressure were collected from the four patient groups. Subsequent one-way ANOVA analysis revealed a consistent trend in which indicators such as R + dp/dtmax, R-dp/dtmax, S + dp/dtmax, S-dp/dtmax, A + dp/dtmax, A-dp/dtmax, C + dp/dtmax, C-dp/dtmax, LV + dp/dtmax, and LV-dp/dtmax decreased with the progression of coronary stenosis. Moreover, certain indicators including S + dp/dtmax, A + dp/dtmax, A-dp/dtmax, C + dp/dtmax, C-dp/dtmax, LV + dp/dtmax, and LV-dp/dtmax showed significant differences between groups, and these differences were statistically significant (*P* < 0.001).

**Figure 2:**
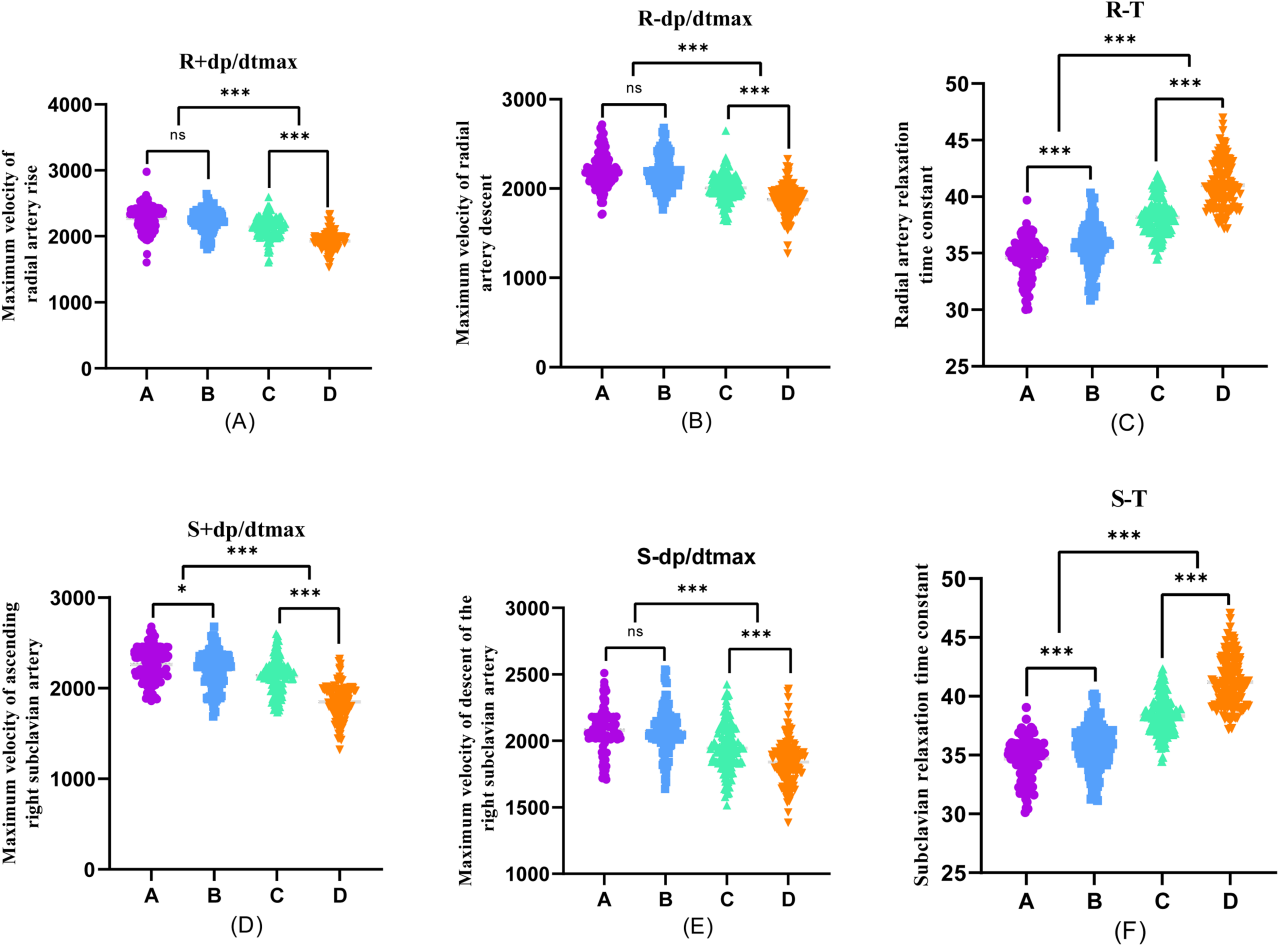
One-way ANOVA analysis of invasive catheterisation data in the radial artery and right subclavian artery. To investigate the relationship between peripheral arterial compliance and left ventricular diastolic function, one-way ANOVA was performed on the data of invasive catheterisation of the radial and right subclavian arteries in four groups of patients, **(A–D)**. **(A)** Coronary artery stenosis free, **(B)** Coronary artery stenosis less than 50%, **(C)** Coronary artery stenosis of 51%–75%, **(D)** Coronary artery stenosis greater than 75%.

**Figure 3:**
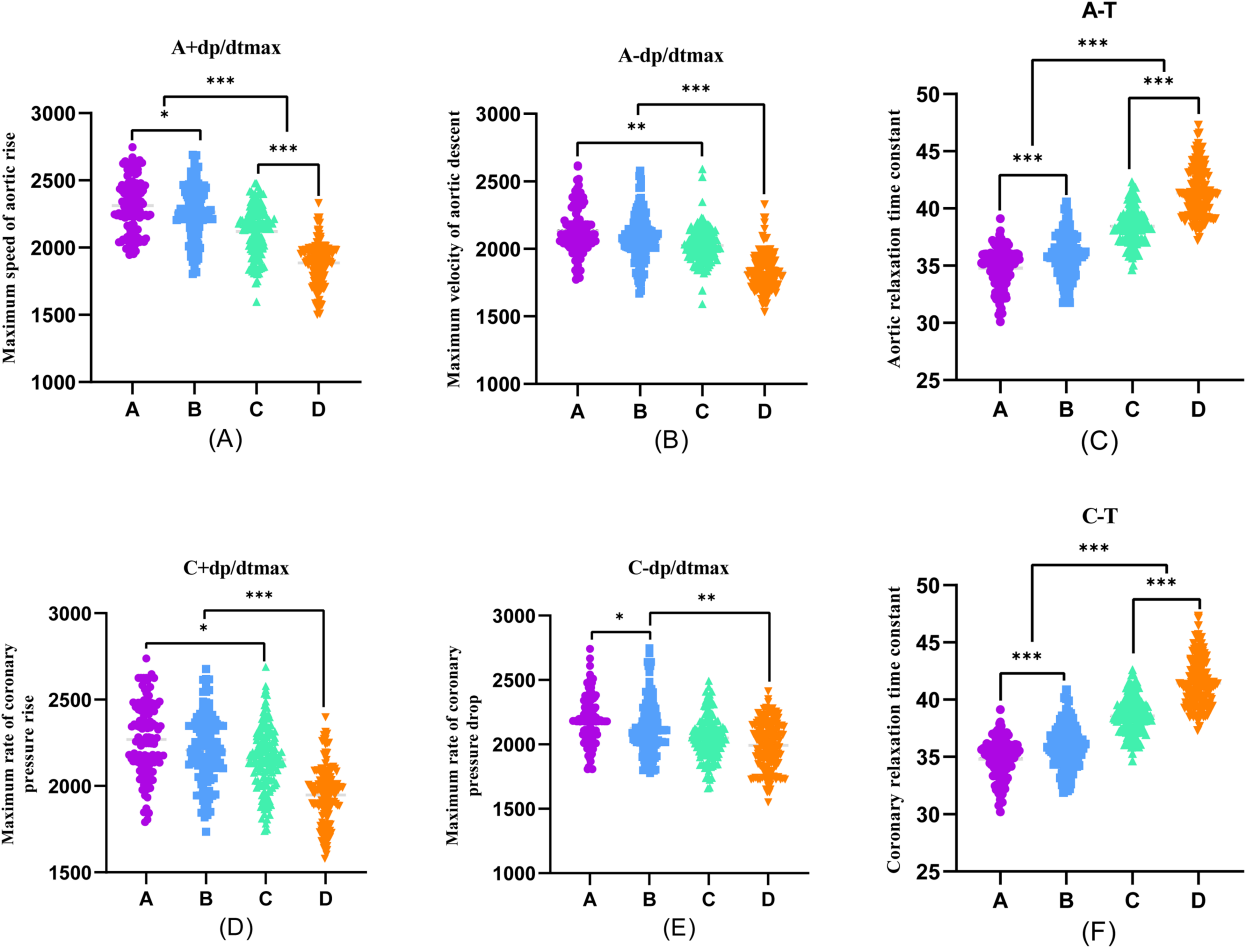
One-way ANOVA analysis of invasive catheterisation data in the aortic and coronary. To investigate the relationship between peripheral arterial compliance and left ventricular diastolic function, one-way ANOVA was performed on aortic and coronary invasive catheterisation data from patients in groups **(A–D)**. **(A)** Coronary artery stenosis free, **(B)** Coronary artery stenosis less than 50%, **(C)** Coronary artery stenosis of 51%–75%, **(D)** Coronary artery stenosis greater than 75%.

**Figure 4:**
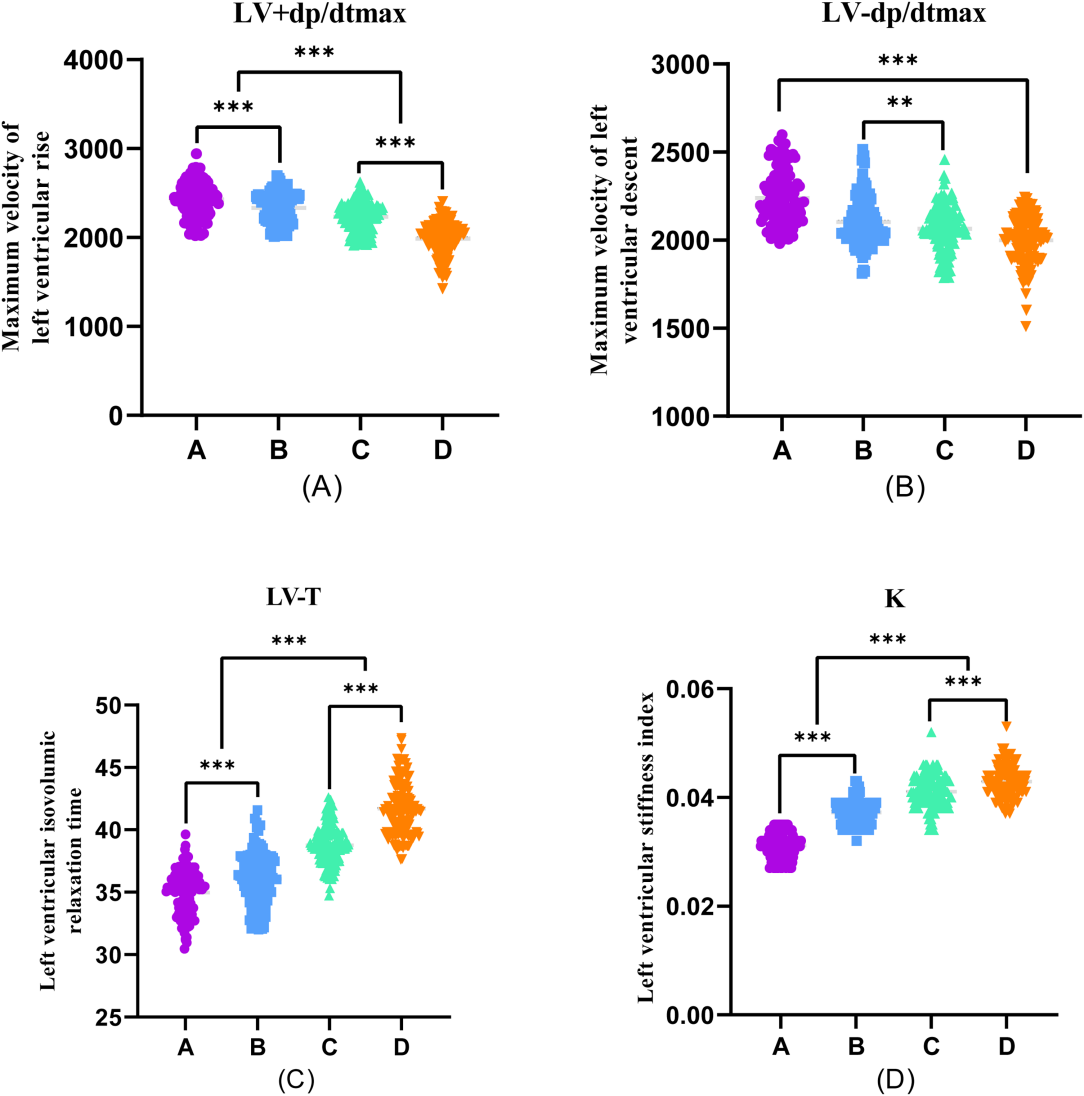
One-way ANOVA analysis of invasive catheterisation data in the left ventricular. To investigate the relationship between peripheral arterial compliance and left ventricular diastolic function, one-way ANOVA was performed on left ventricular invasive catheterisation data from four groups of patients **(A–D)**. **(A)** Coronary artery stenosis free, **(B)** Coronary artery stenosis less than 50%, **(C)** Coronary artery stenosis of 51%–75%, and **(D)** Coronary artery stenosis greater than 75% were collected for invasive catheterisation data.

The differences in R + dp/dtmax, R-dp/dtmax, and S-dp/dtmax were not statistically significant between Groups A and B (*P* > 0.05). However, significant differences emerged between Groups C and D, and these differences were statistically significant (*P* < 0.001). Employing one-way ANOVA to examine the diastolic function of peripheral arteries and the left ventricle revealed significant differences in R-T, S-T, A*-T, C*-T, and LV-T among the four groups. These differences were statistically significant (*P* < 0.001). A comparison of the T values in each group underscored an escalating trend as the degree of coronary artery stenosis increased, signifying a concurrent increase in the T value of the left ventricle and a corresponding decrease in diastolic function (Figures 2–4).

To elucidate the relationship between the diastolic function of individual peripheral arteries and the left ventricle, this study proceeded by correlating the T-values of diastolic indices for each peripheral artery with the T-values of diastolic function indices for the left ventricle.

### Peripheral arterial diastolic function as a predictor of left ventricular diastolic function

A straightforward correlation analysis was conducted between the peripheral arterial T-values of patients suspected of having ACS and their LV-T. The results indicated positive correlations between E/e’, LVEDV, LVESD, LVEDD, CFPWV, DT, Gensini Score, R-T, S-T, A*-T, C*-T, and K values with the LV-T procedure. Conversely, LVEF exhibited a negative correlation with LV-T. Pearson correlation analysis found that each peripheral arterial T-value was closely correlated with LV-T (Table 3).

**Table 3:** Association of peripheral arterial diastolic function with left ventricular diastolic function in patients with suspected ACS (T-value).

Date	r	*P*
E/e’	0.737	<0.01
LVEDV	0.547	<0.01
LVESD	0.282	<0.01
LVEDD	0.349	<0.01
cfpwv	0.720	<0.01
LVEF	−0.528	<0.01
DT	0.505	<0.01
Gensini score	0.731	<0.01
R-T	0.964	<0.01
S-T	0.968	<0.01
A-T	0.969	<0.01
C-T	0.971	<0.01
K	0.595	<0.01

cfPwv, carotid-femoral pulse wave velocity; LVEF, left ventricular ejection fractions; DT, peak E deceleration time; LVEDV, left ventricular end-diastolic volume; LVESD, left ventricular end systolic dimension; LVEDD, left ventricular end-diastolic diameter; E/e’, Ratio of peak early mitral valve diastolic flow to mitral annular motion velocity; R-T, radial artery relaxation time constant; S-T, subclavian relaxation time constant; A-T, aortic relaxation time constant; C-T, Coronary relaxation time constant; LV-T, left ventricular isovolumic relaxation time; K, Left ventricular stiffness index.

**Figure 5:**
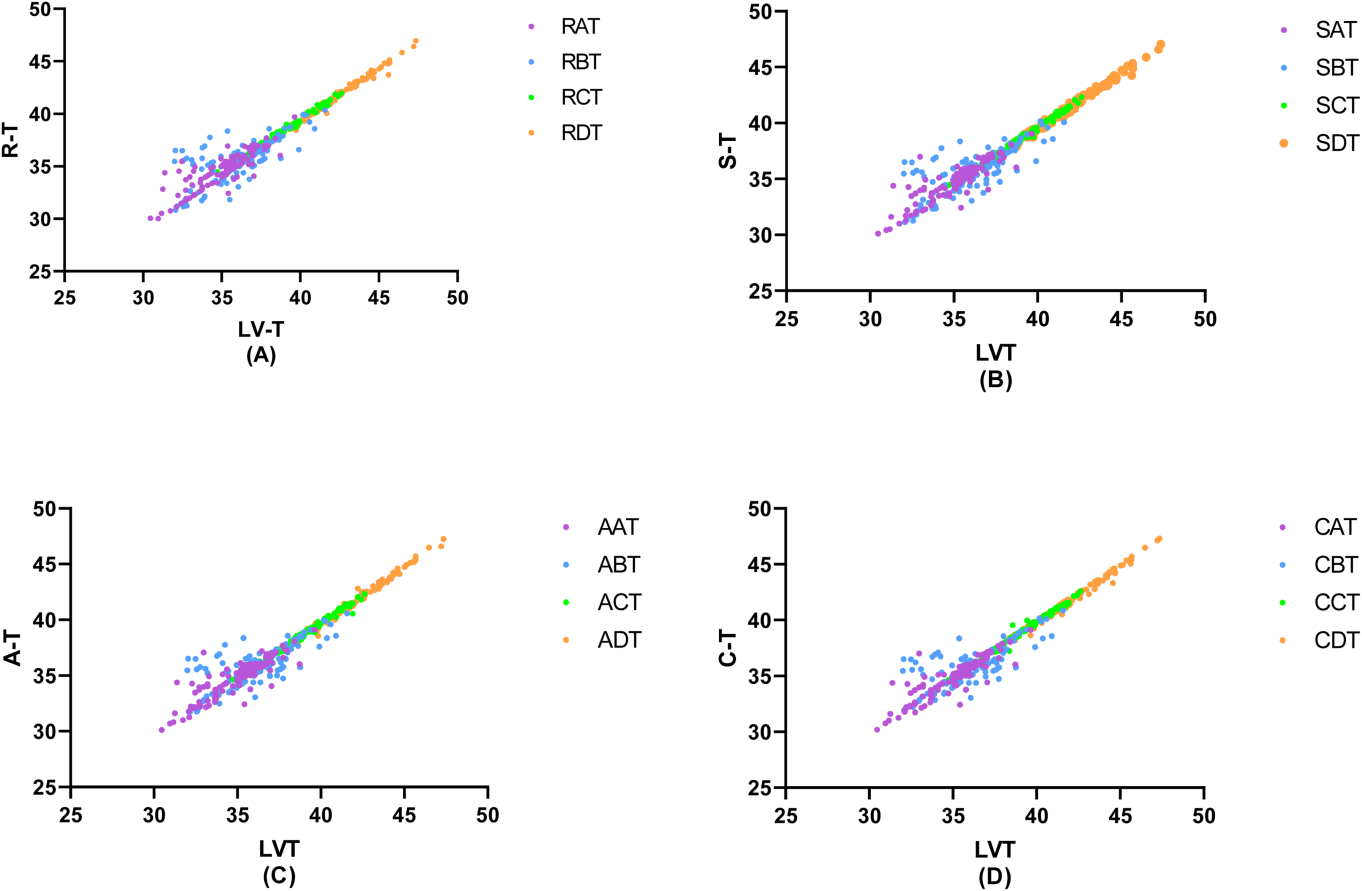
Correlation of invasive peripheral arterial catheterisation data with left ventricular isovolumic relaxation time. In order to study the relationship between left ventricular diastolic function and peripheral arterial compliance in patients with different degrees of coronary artery stenosis, the groups were classified according to the degree of coronary artery stenosis as **(A)** Coronary artery stenosis free, **(B)** Coronary artery stenosis less than 50%, **(C)** Coronary artery stenosis of 51%–75%, **(D)** Coronary artery stenosis greater than 75%, and the values of peripheral arterial compliance in each group were correlated with the left ventricular relaxation time constant, T. The values of peripheral arterial stenosis in each group were correlated with the left ventricular relaxation time constant, T, in each group.

In a detailed examination of how the degree of coronary artery stenosis influences the correlation between peripheral arterial T-values and Lv-T, this study conducted correlation analyses of R-T, S-T, A*-T, and C*-T across different sites within each group and LV-T within each group. The findings revealed significant correlations in various combinations(*p* < 0.01). The investigation unveiled a connection between peripheral arterial T-values and LV-T in each of the four groups (A, B, C, and D). Remarkably, the peripheral arterial T-values in groups C and D showed a particularly strong correlation with the LV-T (Table 4, Figure 5).

**Table 4:** Association of peripheral arterial diastolic function with left ventricular diastolic function in patients with different degrees of coronary artery stenosis (T-value).

			A (*n* = 128)	B (*n* = 135)	C (*n* = 143)	D (*n* = 150)
	LV-T	R-T	0.871	0.741	0.993	0.994
		S-T	0.878	0.759	0.994	0.995
r		A*-T	0.877	0.774	0.994	0.995
		C*-T	0.891	0.781	0.993	0.994
		P	<0.01*	<0.01*	<0.01*	<0.01*

A: Coronary artery stenosis free, B: Coronary artery stenosis less than 50%, C: Coronary artery stenosis of 51–75%, D: Coronary artery stenosis greater than 75%, R-T, radial artery relaxation time constant; S-T, subclavian relaxation time constant; A*-T, aortic relaxation time constant; C*-T, coronary relaxation time constant; LV-T: left ventricular isovolumic relaxation time.

In this investigation, further Linear analysis of peripheral arterial T values and LV T values in groups A, B, C, and D revealed that there was no linear correlation between R-T, S-T, A*-T, C*-T, and LV-T in groups A and B (*P* > 0.05), whereas there was a linear correlation between R-T, S-T, A*-T, C*-T, and LV-T in patients in groups C and D, and the correlation was statistically significant (*P* < 0.001) (Table 5).

**Table 5:** Linear analysis of peripheral arterial T and LV- T in patients with suspected ACS.

	A	B	C	D
R	0.395	0.246	0.210	0.162
S	−0.264	−0.200	0.236	0.277
A*	−0.095	0.168	0.246	0.288
C*	0.927	0.697	0.331	0.270
P	>0.05	>0.05	<0.001	<0.001
95%CI	(−0.64,0.45)	(−0.73,1.06)	(0.02,0.40)	(0.15,0.42)

A: Coronary artery stenosis free, B: Coronary artery stenosis less than 50%, C: Coronary artery stenosis of 51–75%, D: Coronary artery stenosis greater than 75%. Linear analysis of the overall linear equation holds true and is statistically significant in group C patients(*P* < 0.001) y = 0.210R-T + 0.236S-T + 0.246A*-T + 0.331C*-T; Linear analysis of the overall linear equation holds true and is statistically significant in group D patients (*P* < 0.001) y = 0.162R-T + 0.277S-T + 0.288A*-T + 0.270C-T.R, represents the radial artery; S, represents the subclavian artery; A*, represents the principal artery, C*, represents the Coronary artery. 95%CI, confidence interval.

To delve deeper into the impact of radial artery, subclavian artery, and aortic compliance on left ventricular diastolic function, a one-way ANOVA was conducted on the T-values of diastolic function indexes, including R-T, S-T, A*-T, and C*-T, within groups C and D. The results indicated no statistically significant differences in the T-values of diastolic function indexes among the radial artery, subclavian artery, aorta, and coronary artery within the groups, suggesting consistent changes in the diastolic function of peripheral arteries at various sites (*P* > 0.05) (Table 6).

**Table 6:** Diastolic function of arteries at different sites for each group of patients with suspected ACS (T-values).

	R	S	A*	C*	*P*-value	95% CI
A	34.59 ± 1.76^a^	34.69 ± 1.73^a^	34.80 ± 1.70^a^	34.83 ± 1.67^a^	>0.05	(−0.53,0.32)
B	35.55 ± 1.92^a^	35.64 ± 1.88^a^	35.79 ± 1.82^a^	35.87 ± 1.82^a^	>0.05	(−0.54,0.35)
C	38.16 ± 1.58^a^	38.31 ± 1.60^a^	38.44 ± 1.58^a^	38.57 ± 1.60^a^	>0.05	(−0.51,0.22)
D	41.01 ± 2.18^a^	41.19 ± 2.17^a^	41.38 ± 2.19^a^	41.50 ± 2.16^a^	>0.05	(−0.66,0.29)

A: Coronary artery stenosis free, B: Coronary artery stenosis less than 50%, C: Coronary artery stenosis of 51–75%, D: Coronary artery stenosis greater than 75%, values are mean ± standard deviation.

R, represents the radial artery; S, represents the subclavian artery; A*, represents the principal artery; C*, represents the Coronary artery (left side of the heart). The letter ‘^a^’ is used to indicate a significant difference between the groups, while the same letter indicates that the difference between the two groups is not statistically significant. 95%CI: Confidence Interval.

To further explore the impact of the peripheral arterial system, specifically the mean T-value of each peripheral arterial index related to diastolic function, on the left ventricular diastolic function, the current study conducted additional correlation analyses by considering the uniform-T (uT) of the T-value for each peripheral artery within groups C and D.Pearson correlation analysis unveiled a significant correlation between C-uT, D-uT, and LV-T (*P* < 0.01). Further linear analysis of C-uT and D-uT demonstrated a linear correlation between the uT values of both C and D groups and their corresponding LV-T (*P* < 0.05) (Table 7).

**Table 7:** Relevance of uT.

	C	D
uT	0.996	0.997
Linear		
uT	1.004	0.998
P	<0.001	<0.001

C: Coronary artery stenosis of 51–75%, D: Coronary artery stenosis greater than 75%, uT, uniform-T.

## Discussion

Heart failure can be categorized based on left ventricular ejection fraction as HFrEF, HFmrEF and HFpEF (7). Previous research indicates that during myocardial ischemia and hypoxia, changes in diastolic function indexes manifest earlier and more prominently than alterations in systolic function indexes, demonstrating higher sensitivity (15). Despite the early decline in myocardial diastolic function, it does not adversely impact systolic function, leading to the potential oversight of early diastolic function deterioration. Recent studies have established a robust association between left ventricular diastolic dysfunction and the initiation and progression of heart failure, along with an elevated risk of all-cause mortality in patients (16–18). Consequently, early detection or prediction of left ventricular diastolic dysfunction and timely intervention play a crucial role in preventing and ameliorating the incidence of heart failure and mortality in patients.

Non-invasive assessment methods such as echocardiography, cardiac magnetic resonance imaging, and the H2FPEF Score (Heavy, Hypertensive, Atrial Fibrillation, Pulmonary Hypertension, Elder, Filling Pressure) are making their way into clinical practice (19–21). These methods are utilized for early disease screening, among other purposes, but are constrained by their indirect measurement or assessment, leading to less precise and intuitive results compared to invasive tests. The relaxation time constant, detected and calculated through invasive catheterization, stands out as a load-independent measure, serving as the most sensitive and specific indicator for evaluating cardiac and peripheral vascular compliance in patients. Additionally, our prior research has revealed a close association between the degree of coronary atherosclerosis and left ventricular diastolic function (22). This aligns with the findings of a foreign ARIC study, which, over a 13-year follow-up period, observed a close connection between left ventricular diastolic function and coronary arteries in 9,902 study subjects without coronary artery disease (CAD) and heart failure (HF) (23). Studies have also shown that in patients with myocardial infarction without significant coronary artery stenosis, the incidence of heart failure with preserved ejection fraction (HFpEF) is significantly high during long-term follow-up (24). Consequently, exploring the correlation between peripheral arterial compliance, coronary arteries, and left ventricular diastolic function becomes crucial for the early identification or prediction of left ventricular diastolic dysfunction in patients. Many studies have primarily concentrated on individually assessing or predicting myocardial diastolic function or peripheral vascular compliance. Fewer investigations have explored the interrelation between peripheral arterial compliance and left ventricular diastolic function. In this study, coronary angiography was conducted through radial artery puncture, and intraoperative pressure curves of the radial artery, right subclavian artery, aorta, left coronary artery, and left ventricle were measured. Subsequently, T-values were calculated and subjected to statistical analysis to elucidate the connection between peripheral arteries and the left ventricle. The aim is to enhance the accuracy and predictability of predicting heart failure onset and progression, ultimately reducing rates of rehospitalization and all-cause mortality in patients.

Arterial stiffness is recognized to play a role in the initiation, advancement, and unfavorable prognosis of heart failure, with indications of altered arterial stiffness evident in early-stage heart failure patients (25). This stiffness is reflective of the elasticity or compliance of arterial walls, and studies have demonstrated arterial compliance as an independent predictor of diastolic insufficiency (26). In a study involving 385 patients with heart failure with HFpEF, an exercise trial revealed a robust association between alterations in arterial stiffness and left ventricular hemodynamics, contributing to changes in left ventricular diastolic function (27). Ventricular-arterial stiffness constitutes a crucial element in the pathophysiology of modified ventricular diastolic function. The increase in arterial stiffness intensifies the cardiac afterload and simultaneously reduces the coronary perfusion during diastole. This cascade effect leads to the gradual decompensation of left ventricular diastolic function, initiating a continuous decline and culminating in the progression to irreversible myocardial fibrosis and heart failure (28). Therefore, this study aimed to explore the correlation between peripheral arteries and the left ventricle, enabling the early detection or prediction of the onset of left ventricular diastolic dysfunction. In this study, as the degree of coronary artery stenosis increased, the peripheral arterial compliance decreased accordingly, and the decrease in peripheral arterial compliance in the experimental group patients was significantly less than that in Group A (control group) patients, which is consistent with the findings of Chirinos et al. (29) and Song et al. (30). This indicates a close association between peripheral arterial compliance and left ventricular diastolic function.

This study statistically analyzed the T-values of peripheral arteries, coronary arteries, and the left ventricle in patients from Groups A, B, C, and D. In cases where the coronary artery stenosis was less than 50%, the correlation between the T-values of peripheral arteries and the left ventricle showed slight variations. We hypothesized that this observation might be due to the stenosis being less than 50%, which leads to a delayed change in peripheral arterial compliance before significant physiological adaptation occurs. However, when the degree of coronary artery stenosis exceeded 50%, changes in peripheral arterial compliance consistently mirrored changes in the patient's left ventricular diastolic function, declining with an increase in the Gensini score. This pattern is consistent with the results of a study involving 103 patients with peripheral arterial disease (PAD) who had no cardiac symptoms or known coronary artery disease (31).

In the investigation, a one-way ANOVA of peripheral arterial T-values in groups C and D showed no statistically significant difference in compliance index T-values across various sections of peripheral arteries between the groups (*P* > 0.05). Subsequently, the mean uT was computed for the peripheral arteries in this segment of the study for each patient. One-way ANOVA, Pearson's analysis, and LV-T were then conducted. Linear analysis revealed a high degree of consistency between changes in peripheral arterial compliance and left ventricular diastolic function when the degree of coronary stenosis exceeded 50% (*P* < 0.01).

## Conclusion

In this investigation, a comparison of changes in peripheral arterial compliance and left ventricular diastolic function across patients with varying degrees of coronary artery stenosis revealed a correlation between a higher degree of coronary artery stenosis and worsened left ventricular diastolic function. Additionally, it was observed that peripheral arterial compliance undergoes changes corresponding to the increase in the degree of coronary artery stenosis. Notably, when the degree of coronary artery stenosis surpasses 50%, alterations in peripheral arterial compliance become closely associated with shifts in left ventricular diastolic function. This discovery offers novel clinical insights into the onset and progression of heart failure with preserved ejection fraction. It has the potential to enhance our understanding of, and ability to identify, the evolving alterations in left ventricular diastolic function among patients. This holds significant clinical implications for the early diagnosis of HFpEF or for impeding its onset and progression.

## Limitations

The participants of this study were exclusively from Fengxian District Central Hospital, which made it a single-center exploratory study rather than a nationwide or multicenter collaborative project. Due to differences in operational habits and physiological structures, some errors in the data collection process are inevitable. Additionally, this investigation relies on retrospective data, emphasizing the necessity for forthcoming prospective studies involving individuals with suspected ACS.

## Data Availability

The original contributions presented in the study are included in the article/Supplementary Material, further inquiries can be directed to the corresponding author.
