# Supplementary material for: Predictive value of percutaneous peripheral arterial compliance T in left ventricular diastolic function with coronary artery disease
Source: Front Cardiovasc Med. 2024 Nov 8;11:1366072. doi: 10.3389/fcvm.2024.1366072 (PMC11582064; doi:10.3389/fcvm.2024.1366072)
Supplement: Supplementary file 1 [file Datasheet1.pdf]

Supplementary Material

Supplementary Tables

Table1S Datamaterial on invasive catheter pressure curves in patients with suspected ACS

	A<br>（n=128）	B<br>（n=135）	C<br>（n=143）	D<br>（n=150）	P value
R+dp/dtmax(mmHg/s)	2273.35±193.52[a]	2182.20±182.52[a]	2143.69±154.19[b]	1932.24±131.79[c]	＜0.001
R-dp/dtmax(mmHg/s)	2205.90±190.31[a]	2183.81±190.31[a]	2010.66±150.84[b]	1875.23±160.54[c]	＜0.001
R-T	34.59±1.76[a]	35.55±1.92[b]	38.16±1.58[c]	41.01±2.18[d]	＜0.001
S+dp/dtmax(mmHg/s)	2261.34±185.69[a]	2206.73±195.22[b]	2133.76±182.38[c]	1850.70±180.39[d]	＜0.001
S-dp/dtmax(mmHg/s)	2081.30±155.12[a]	2069.03±155.20[a]	1948.08±173.37[b]	1842.97±167.65[c]	＜0.001
S-T	34.69±1.73[a]	35.64±1.88[b]	38.31±1.60[c]	41.19±2.17[d]	＜0.001
A+dp/dtmax(mmHg/s)	2313.56±198.73[a]	2264.99±193.49[b]	2121.75±183.32[c]	1887.10±154.21[d]	＜0.001
A-dp/dtmax(mmHg/s)	2135.79±167.60[a]	2082.31±165.98[b]	2026.12±128.68[c]	1830.71±134.86[d]	＜0.001
A-T	34.80±1.70[a]	35.79±1.82[b]	38.44±1.58[c]	41.38±2.19[d]	＜0.001
C+dp/dtmax(mmHg/s)	2270.38±210.99[a]	2205.33±193.80[b]	2153.97±179.60[c]	1948.66±166.91[d]	＜0.001
C-dp/dtmax(mmHg/s)	2185.73±183.20[a]	2126.84±205.61[b]	2051.75±167.92[c]	1993.64±185.61[d]	＜0.001
C-T	34.83±1.67[a]	35.87±1.82[b]	38.57±1.60[c]	41.50±2.16[d]	＜0.001
LV+dp/dtmax(mmHg/s)	2442.27±197.72[a]	2336.43±166.52[b]	2233.43±162.23[c]	1988.96±182.37[d]	＜0.001
LV-dp/dtmax(mmHg/s)	2240.27±153.28[a]	2104.53±133.73[b]	2066.33±129.15[c]	2001.17±136.77[d]	＜0.001
LV-T	35.00±1.77[a]	36.08±2.09[b]	38.77±1.58[c]	41.71±2.08[d]	＜0.001
K	0.031±0.002[a]	0.037±0.002[b]	0.041±0.003[c]	0.043±0.003[d]	＜0.001

A: Coronary artery stenosis free, B: Coronary artery stenosis less than 50%, C: Coronary artery stenosis of 51-75% , D: Coronary artery stenosis greater than 75% . Values are mean ± standard

deviation. R+dp/dtmax: Maximum velocity of radial artery rise, R-dp/dtmax: Maximum velocity of radial artery descent, R-T: Radial artery relaxation time constant, S+dp/dtmax: Maximum velocity of ascending right subclavian artery, S-dp/dtmax: Maximum velocity of descent of the right subclavian artery, S-T: Subclavian relaxation time constant, A+dp/dtmax:Maximum speed of aortic rise, A-dp/dtmax: Maximum velocity of aortic descent, A-T: Aortic relaxation time constant, C+dp/dtmax: Maximum rate of coronary pressure rise, C-dp/dtmax: Maximum rate of coronary pressure drop, C-T: Coronary relaxation time constant, LV+dp/dtmax: Maximum velocity of left ventricular rise, LV-dp/dtmax: Maximum velocity of left ventricular descent, LV-T: Left ventricular isovolumic relaxation time, K: Left ventricular stiffness index. [a,b,c,d] are markers of difference between the groups, the same letter indicates that the difference between the two groups is not statistically significant, and different letters indicate that the difference is statistically significan

1.1 Supplementary Figures

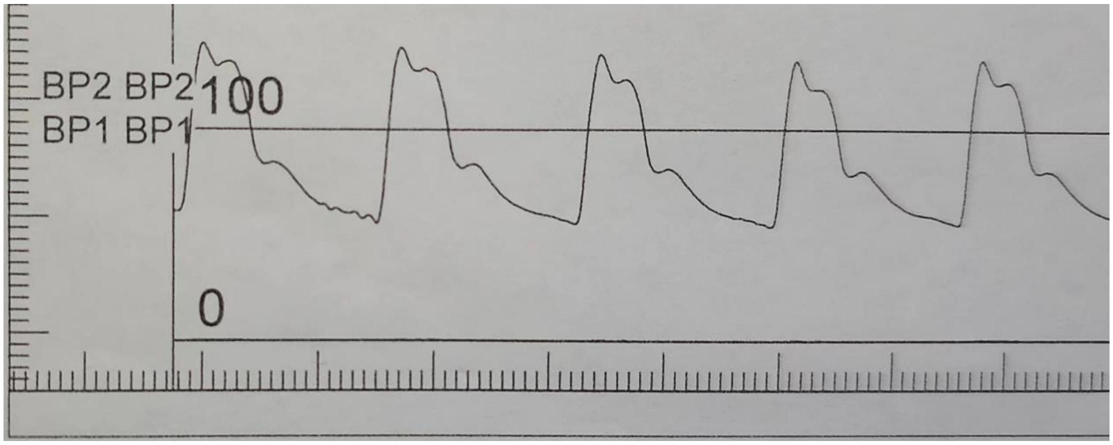

Supplementary Figure 1 Radial Artery Pressure Curve Schematic

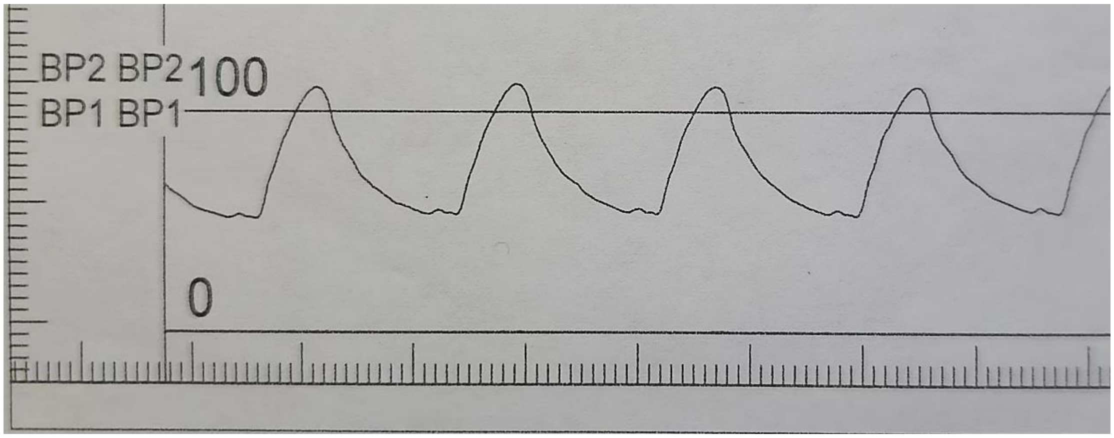

Supplementary Figure 2 Right Subclavian Artery Pressure Curve Schematic

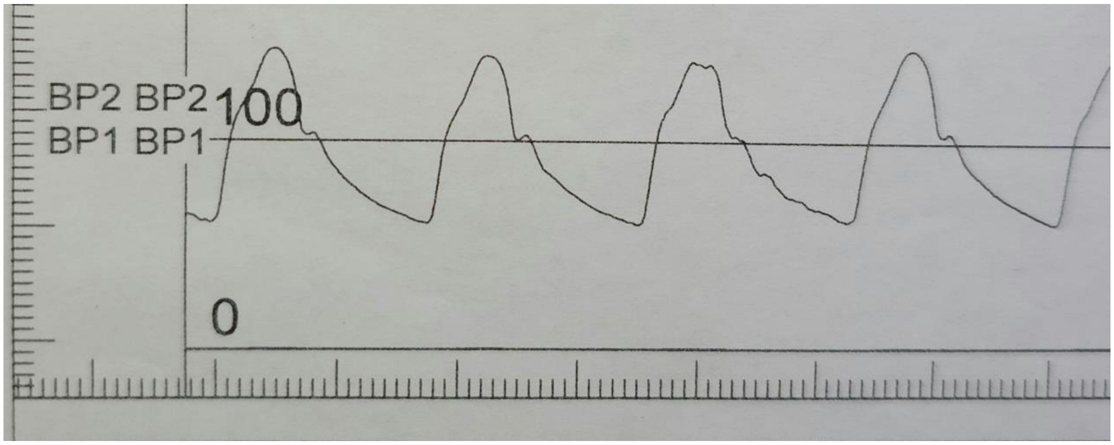

Supplementary Figure 3 Artery Pressure Curve Schematic

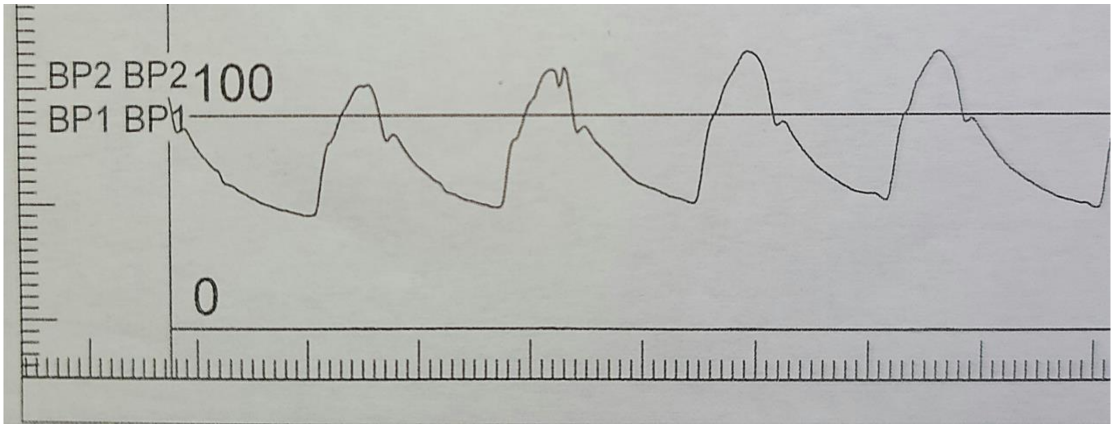

Supplementary Figure 4 Coronary Pressure Curve Schematic

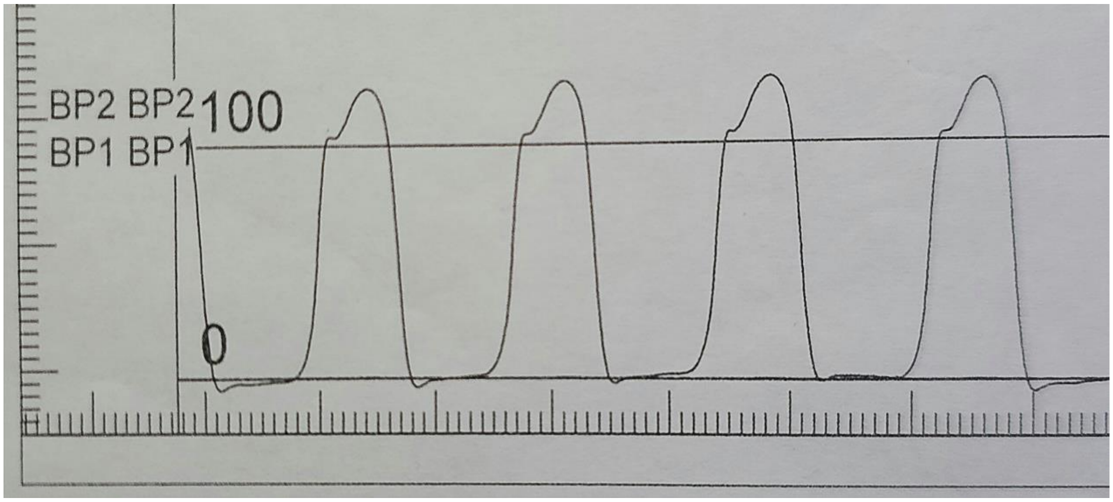

Supplementary Figure 5 Left Ventricular Pressure Curve Schematic
